# DNMT1 recruited by EZH2-mediated silencing of miR-484 contributes to the malignancy of cervical cancer cells through MMP14 and HNF1A

**DOI:** 10.1186/s13148-019-0786-y

**Published:** 2019-12-07

**Authors:** Yang Hu, Fuxia Wu, Yankun Liu, Qian Zhao, Hua Tang

**Affiliations:** 1 Tianjin Life Science Research Center, Tianjin Laboratory of Inflammation Biology, Collaborative Innovation Center of Tianjin for Medical Epigenetics, Department of Pathogen Biology, Basic Medical School, Tianjin Medical University, 22 Qi-Xiang-Tai Road, Tianjin, 300070 China; 2 The Cancer Institute, Tangshan People’s Hospital, Tangshan, 063001 China; 3 Department of Cell Biology, Tianjin Medical University, Tianjin, 300070 China

**Keywords:** DNA methylation, Histone modification, miR-484, DNMT1, Cervical cancer

## Abstract

**Background:**

Emerging evidence indicates that dysregulation of microRNAs (miRNAs) contributes to cervical cancer (CC) tumorigenesis and development. Previous work showed that miR-484 which regulated the EMT process was obviously downregulated in CC. However, little is known about the precise mechanism.

**Results:**

We found that the deficiency of EZH2-recruited DNA methyltransferases DNMT1 reduced the CpG methylation of miR-484 promoter and then increased the miR-484 expression. Furthermore, the cell membrane-bound matrix metalloproteinase (MMP14) and the hepatocyte nuclear factor 1A (HNF1A) were found to be downregulated by miR-484. miR-484 repressed the expression of MMP14 and HNF1A inhibiting CC growth and metastasis in vitro and in vivo. Upregulation of MMP14 and HNF1A promotes the CC cell adhesion and EMT, all of which contribute to cell motility and metastasis. Moreover, miR-484 negatively regulates the WNT/MAPK and TNF signaling pathway by downregulating HNF1A and MMP14 respectively. Thus, miR-484, who is downregulated by DNMT1-mediated hypermethylation in its promoter, functions as a tumor suppressor by inhibiting MMP14 and HNF1A expression in CC.

**Conclusion:**

Our finding characterizes miR-484 as a key suppressive regulator in CC metastasis and reveals a DNMT1-mediated epigenetic mechanism for miR-484 silencing, expanding our understanding of the molecular mechanism underlying CC progression and metastasis.

**Graphical abstract:**

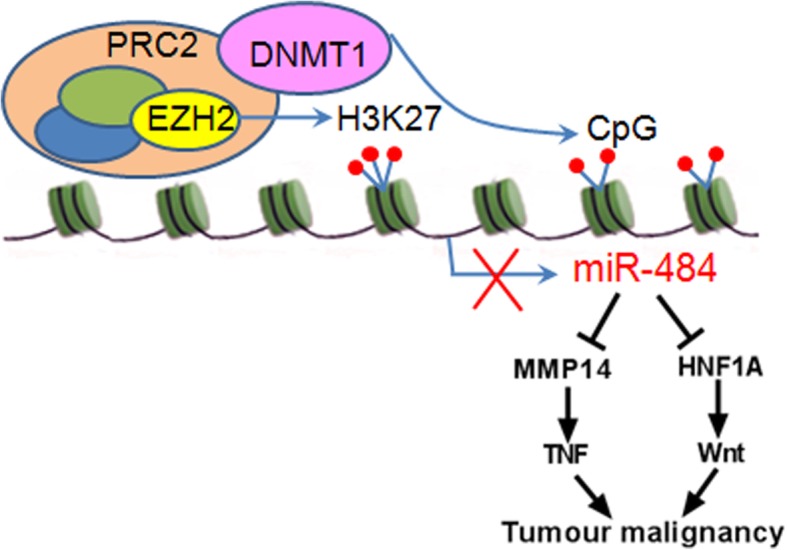

## Introduction

Cervical cancer (CC) is the second most common cancer among women worldwide, accounts for a large proportion of cancer-associated mortalities [1]. Although the decline in CC incidence has largely been attributed to widespread use of cytology screening, invasive CC still threatens the lives of women. Thus, it is urgent to identify specific molecules and markers that contribute to understanding cervical carcinogenesis and ascertaining diagnostic and treatment strategies. Recently, researchers have focused on the effect of miRNAs on CC and a lot of miRNAs were found to play great importance in the initiation and development of CC [2–4].

However, little is known about the causes of the widely differential expression of miRNAs between cancer and normal cells [5, 6]. In cancer, CpG islands of the promoter are commonly hypermethylated, and the methylation is often associated with repression of the target gene [7]. In fact, approximately 20% of all miRNAs are embedded within CpG islands [8]. DNA methylation plays a key role in the silencing of numerous miRNAs encoding genes, suggesting the existence of tumor-suppressive miRNAs that are epigenetically downregulated in CC. In previous work, we found that miR-484 was downregulated in cervical cancer tissues and cell lines compared with their matched non-cancerous tissues or normal cervical keratinocytes cells. However, the mechanism by which miR-484 is downregulated is relatively unknown. DNA methyltransferases enzymes (DNMTs) are directly responsible for the hypermethylation of CpG islands in promoters. However, DNA methylation is closely associated with histone modification due to the crosstalk mediated by the methyl-CpG-binding domain proteins (MBDs) [9]. Thus, methylation-associated molecules, including DNMTs, MBDs, histone deacetylases (HDACs), histone methyltransferases (HMTs), and lysine demethylase (KDMs), may play important roles in the silencing of the miR-484 gene.

Increasing evidence suggests that miRNAs participate in nearly every step of the pathogenesis of cancer [2]. Our previous work showed that miR-484 inhibited cell proliferation and the epithelial–mesenchymal transition (EMT) process [10]. It is well known that EMT can disrupt intercellular contacts, enhance cell motility, and facilitate the release of cancer cells from the primary tumor [2]. In addition, the metastatic mechanism also includes the interaction between tumor cells and microenvironment at secondary sites, such as cell–matrix adhesion. However, the relationship between miR-484 and cell adhesion is still not clear. The cell membrane-bound matrix metalloproteinase 14 (MMP14), an ECM remodeling protein, contributes to invasion, metastasis, and angiogenesis through ECM degradation, is an important cell adhesion molecule involved in the EMT process [11].

The hepatocyte nuclear factor 1 (HNF-1) transcription factor family includes HNF-1 (also known as HNF-1α), and variant isoforms of the HNF-1 (vHNF-1) (also known as HNF-1β). These transcription factors are expressed in a different spatio-temporal manner in the yolk sac endoderm, and in the developing kidney, liver, and pancreas [12]. In normal tissues, HNF-1 is expressed in epithelial cells of the urogenital tract, liver, pancreas, gut, and lung regulating epithelia-specific gene expression [13, 14]. In tumors, mutations and epigenetic inactivation of the HNF-1β gene has been shown to be involved in the development of several cancers [15–17]. Recently, it has been reported that the expression of HNF-1β in cervical carcinoma is mostly restricted to adenocarcinomas and can be used as an auxiliary adenocarcinoma marker in the differential diagnosis of poorly differentiated cervical carcinomas [18]. However, the expression and precise role of HNF-1α (HNF1A) in carcinogenesis as well as the importance of molecular targeting of this protein for therapeutic purposes remains unknown.

Here, we performed qRT-PCR and genomic bisulfite sequencing to investigate the epigenetic silencing of miR-484 in clinical CC samples and the cell lines. We found that DNMT1 recruited by EZH2-mediated hypermethylation of the miR-484 promoter. Moreover, MMP14 and HNF1A were identified as functional targets of miR-484. miR-484 suppressed cell adhesion and the EMT process through negatively regulating the WNT/MAPK and TNF signaling pathway in CC cells. Collectively, the present work provides the first evidence of the coordination of methylation modulated miR-484 and MMP14/HNF1A in the regulation of the cell adhesion, EMT, and the β1-integrin pathway during cervical cancer carcinogenesis.

## Materials and methods

### Human cervical cancer tissue specimens and cell lines

CC tissues and the paired adjacent non-tumor cervical tissues were obtained from the cancer center of Sun Yat-sen University. The diagnose was evaluated by pathological analysis. Written informed consent was obtained from each patient and ethics approval for this work was granted by the Ethics Committee of Sun Yat-Sen University. Cell culture and determination of growth rates were according to ref. [19]. S12, an immortalized human cervical keratinocyte cell line, was a kind gift from Prof. Wang (Tongji Hospital, Tongji Medical College, Huazhong University of Science and Technology, Wuhan, Hubei, China). All the cells were maintained in a humidified incubator with 5% carbon dioxide (CO_2_) at 37 °C.

### Vector construction

The pcDNA3/pri-miR-484 (pri-miR-484) expression vector and ASO-miR-484 were obtained from our previous work [10]. pcDNA3-Flag-MMP14/HNF1A plasmids (MMP14/HNF1A) were constructed to intensify MMP14/HNF1A expression, and pSilencer-MMP14/HNF1A (shR-MMP14/HNF1A) was synthesized to silence the endogenous MMP14/HNF1A. The EGFP reporter vector pEGFP-MMP14/HNF1A 3′-UTR contained the binding sites of miR-484 to MMP14/HNF1A 3′-UTR. The vector pEGFP-MMP14/HNF1A-3′-UTR-mut contained the mutated binding sites of miR-484 to MMP14/HNF1A 3′-UTR. The details of the methods constructing these vectors and the primers are in ref. [10]. All insertions described above were verified by DNA sequencing. The DNMT1, DNMT3A, DNMT3B, KDM2A, KDM4A, and KDM4B siRNAs were purchased from GenePharma (Suzhou, China). All the primers are shown in Additional file 1: Table S1.

### Prediction of miRNA targets

miR-484 predicted targets were retrieved from TargetScan (http://genes.mit.edu/tscan/targetscanS.html), miRecords (http://c1.accurascience.com/miRecords/), and PITA (https://genie.weizmann.ac.il/pubs/mir07/), and the binding site predictions were performed using RNAhybrid (http://bibiserv.techfak.uni-bielefeld.de/rnahybrid/).

### Prediction of the miR-484 promoter and the CpG islands

Using the Promoter 2.0 Prediction Server (http://www.cbs.dtu.dk/services/Promoter/) and Promoter Scan (http://www-bimas.cit.nih.gov/molbio/proscan/), we identified the overlapping region as the putative promoter of miR-484. We inserted this region upstream of the reporter gene in the pGL3-basic/luciferase vector and tested the luciferase activity following the instructions provided by the Dual-Luciferase Reporter Assay system (Promega, USA). We also used MethPrimer (http://www.urogene.org/methprimer/) to predict the CpG islands in the promoter region.

### Genomic bisulfite sequencing

For the demethylation experiments, cells were treated with 5 μmol/L 5-aza-2-deoxy-cytidine (5-Aza-CdR, Sigma) for 72 h, and the drug and medium were replaced every 24 h. Cells treated with DMSO were used as a control. Genomic DNA was extracted using the All Prep DNA/RNA Kit (Qiagen). One microgram of total genomic DNA was treated with sodium bisulfite according to the manufacturer’s instructions provided with the EpiTect Kit (Qiagen). The CpG islands were amplified from the bisulfite-converted DNA by PCR, and the PCR products were cloned and sequenced.

### RNA isolation and reverse transcription quantity (RT-qPCR)

Extraction of total RNA from cells was performed using the TRIzol reagent (Invitrogen, CA) following the manufacturer’s instructions. The total RNA concentration was determined using a NanoDrop Lite (Thermo Fisher Scientific, 2000c). The SYBR Premix Ex Taq Kit (TaKaRa, Shiga, Japan) was used according to the manufacturer’s instructions, and RT-qPCR was performed and analyzed using the iQ5 Detection System (Bio-Rad, Hercules, CA, USA). The method of reverse transcription quantity (RT-qPCR) has been described in ref. [9]. The primers for RT and PCR are provided in Additional file 1: Table S1.

### Cell adhesion assay

Cell–cell adhesion assay and cell–matrix adhesion assay were according to the method in ref. [10]. For the cell–cell adhesion assay, after centrifugation, the treated cells were resuspended at a final concentration of 5 × 10^5^ cells/mL in calcium-free, suspension-modified Eagle’s medium in the absence of serum and with or without cell adhesion inhibitor peptide (cyclic RGDfv, 100 μM, Nanjing Peptide Biotech Ltd, Nanjing, China), and then, 1 × 10^6^ cells were maintained in suspension on 10% poly-2-hydroxyethyl methacrylate-coated (poly-HEMA, Sigma, St. Louis, MO, USA) six-well plates to prevent cell attachment to the substrate. Cells on the substrate in at least three fields per well were imaged after 24 h of culture using an Olympus IX 71 (Tokyo, Japan). Cell aggregates were counted based on the number of cells per aggregate: 24 h (> 20 cells).

For the cell–matrix adhesion assay, the 96-well plates were coated overnight with 10 μg/mL fibronectin (FN) (Solarbio, Shanghai, China) and 200 μg/mL Matrigel (M) at 4 °C and were blocked with 1% (w/v) bovine serum albumin. FN and M were used to simulate cell adhesion to matrix. Cells were suspended in complete medium with or without RGDfv (100 μM) and seeded on the 96-well plates at a density of 2 × 10^4^ per well in triplicate, allowed to adhere at 37 °C for 30, 60, and 90 min, and were then washed three times with phosphate-buffered saline. The cells were fixed with 4% (w/v) paraformaldehyde, stained with 0.5% (w/v) crystal violet for 10 min, and then, the attached cells were lysed with 30% (v/v) glacial acetic acid for 15 min; absorbance at 570 nm was then measured.

### Cell migration and invasion assays

The methods of cell migration and invasion assays have been described in our previous work [10].

### Western blot analysis

The detailed procedures for Western blot were described in the previous study [10]. The primary antibodies used in this study including EZH2, histone 3, MMP14, HNF1A, β-integrin, E-cadherin, vimentin, Dvl1, Wnt2, TNF-R1, FAS, TNF-a, and GAPDH, which were obtained from Saier Co. (Tianjin, China) and Wanleibio Co. (Shenyang, China). The secondary goat anti-rabbit or anti-mouse antibodies were purchased from Sigma.

### Chromatin immunoprecipitation (ChIP) assay

ChIP assays were performed using an EZ-Magna ChIP kit (Millipore, USA), according to the manufacturer’s instructions. Cells were fixed with 1% formaldehyde to crosslink; then, nucleoprotein complexes were sheared to 200–500 bases in length with sonication and then immunoprecipitated with anti-EZH2 or anti-H3K27me3 (Millipore, USA) antibodies overnight at 4 °C. PCR and real-time PCR were used to detect the enrichment of DNA fragments in the putative binding sites of the miR-484 upstream coding region. The primers used to detect the miR-484 upstream sequence were provided in Additional file 1: Table S1. For the real-time PCR analysis, the percentage of bound DNA enrichment was quantified related to input.

### Co-immunoprecipitation (Co-IP) assay

At 48 h post-transfection, CC cells were lysed with RIPA lysis buffer and protein extracts were subsequently incubated using anti-EZH2 at 4 °C for 16 h. The protein antibody complexes were precipitated by incubating the lysates with Bioepitope R protein G/A agarose IP reagent beads (Bioworld Technology, Louis, CA) for 6 h under constant rotation at 4 °C. After incubation, the precipitated proteins were detected by processed for Western blot.

### Xenograft tumor formation assay

HeLa cells were transfected with pri-miR-484 or pcDNA3 and passaged for four generations with culture media containing 500 μg/mL G418. Cells were then harvested, and about 1 × 10^6^ cells, resuspended in 100 μL of serum-free RPMI 1640 culture medium, and were injected subcutaneously into the flanks of the nude mice. Tumor size was measured every 3 days beginning on day 8 after the injection. The tumor volume was calculated as follows: length × width^2^ × 1/2. All mice were sacrificed on day 20 post-injection. The tumors were isolated from the mice and stored at − 80 °C. All studies were performed under American Association for the Accreditation of Laboratory Animal Care guidelines for humane treatment of animals and adhered to national and international standards.

### Statistical analyses

All analyses were performed using SPSS 19 for Windows (SPSS Inc., Chicago, IL, USA) and GraphPad Prism 5 for Windows (GraphPad Software Inc., San Diego, CA, USA). All the data are presented as the mean ± SD. Each experiment was performed at least three times, and the analysis was performed using paired *t* test. *p* ≤ 0.05 was considered statistically significant (*p < 0.05, **p < 0.01, ***p < 0.001).

## Results

### miR-484 is hypermethylated and silenced in CC tissues and cells

**Fig. 1:**
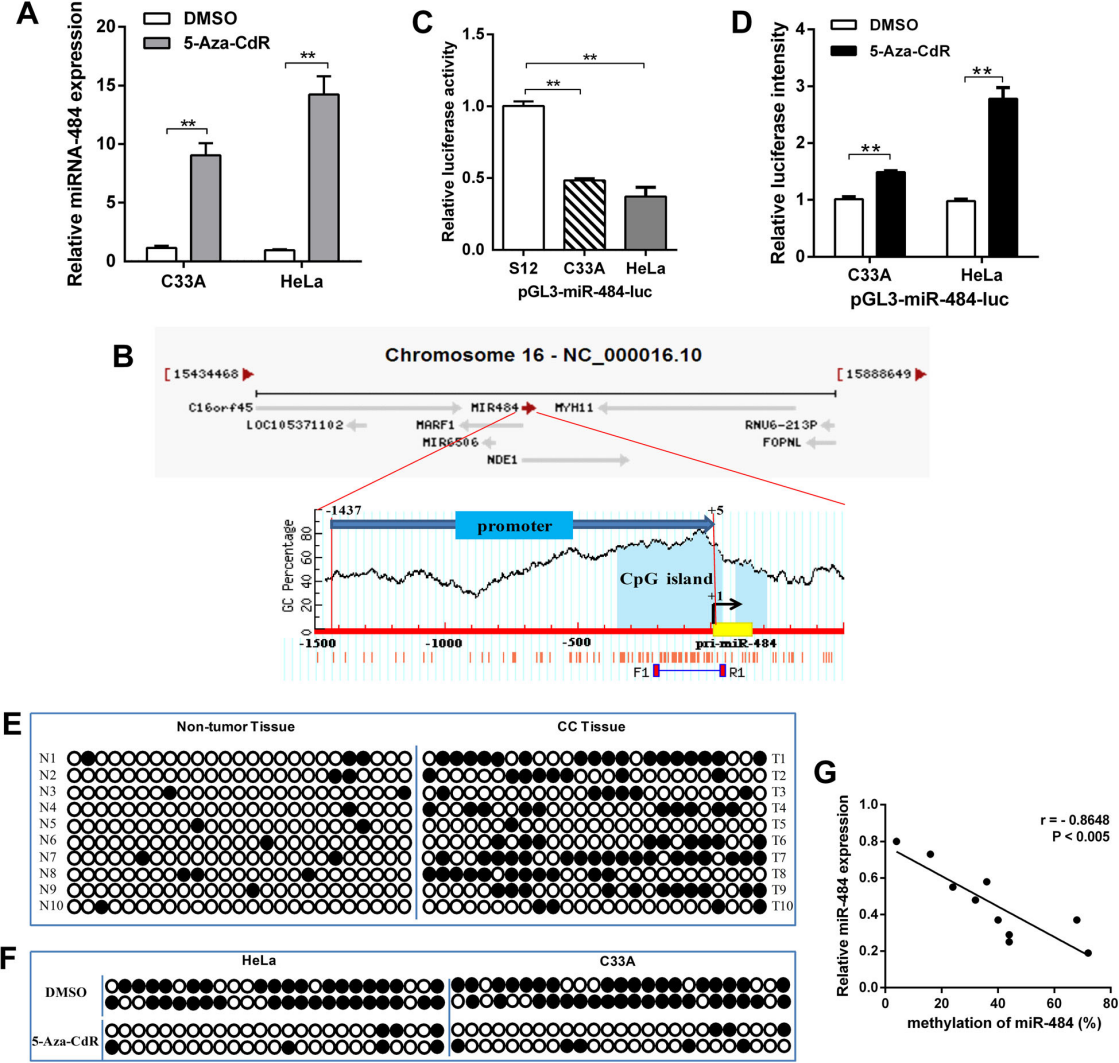
Promoter DNA hypermethylation mediates the downregulation of miR-484 expression in CC. **a** The mRNA level of miR-484 in CC cell lines after treatment with 5-Aza-CdR was measured by RT-qPCR. **b** The diagram shows the promoter region of the miR-484 gene and the CpG island located within this region. The red vertical bar represents the CpG sites. **c** and **d** Luciferase reporter system was used to detect the promoter activity of miR-484 in CC cell lines (**c**) and after 5-Aza-CdR treatment (**d**). **e** genomic bisulfite sequencing was performed to determine the methylation status of the miR-484 promoter in 10 pairs of CC tissues (T1-T10). **f** genomic bisulfite sequencing was performed to determine the methylation status of the miR-484 promoter in CC cell lines after 5-Aza-CdR treatment. The black circle indicates methylated CpG loci and the white circle indicates unmethylated CpG loci. **g** Scatter plots showing miR-484 expression compared with methylation. Error bars in **a**, **c**, and **d** indicate the mean ± SD of three independent experiments. **p < 0.01

In previous work, we examined the expression of miR-484 in 20 pairs of cervical cancer tissues and 6 cervical cancer cell lines by RT-qPCR. The results showed that miR-484 was generally downregulated both in vivo and in vitro [10]. To demonstrate whether DNA methylation results to the downregulated of miR-484 in CC, we treated HeLa and C33A cells with 5-Aza-CdR, which is frequently used to induce demethylation. Next, we examined the expression level of miR-484 by RT-qPCR. The results showed that miR-484 was significantly upregulated after treated with 5-Aza-CdR (Fig. 1a).

To verify the effect of DNA methylation on miR-484 expression, we cloned a fragment with promoter activity (− 1437 to + 5 upstream of miR-484) (Additional file 1: Figure S1) into the pGL3-Basic vector, and we found a CpG island harboring 25 CpG dinucleotides (− 218 to + 5) in this promoter region (Fig. 1b). The luciferase reporter assay revealed that the promoter activity of miR-484 in CC cell lines was lower than that in an immortalized normal human cervical epithelial cell line (S12), and 5-Aza-CdR treatment restored its activity (Fig. 1c and d). Next, genomic bisulfite sequencing was performed to determine the methylation status of the miR-484 promoter in 10 pairs of CC tissues (T1–T10) and cell lines. The results revealed that the methylation level was higher in CC tissues than in normal tissues (Fig. 1e). Meanwhile, miR-484 was highly methylated in HeLa and C33A cells, and the methylation level decreased after 5-Aza-CdR treatment (Fig. 1f). The relationship between methylation and expression can be demonstrated by analyzing the correlation between the genomic DNA and RNA isolated from the same patient. Spearman’s rank correlation analysis revealed an inverse correlation between methylation and the expression of miR-484 (Fig. 1g). These results suggest that miR-484 is epigenetically downregulated in CC.

### EZH2-recruited DNMT1 mediated DNA hypermethylation, thereby inducing miR-484 silencing

**Fig. 2:**
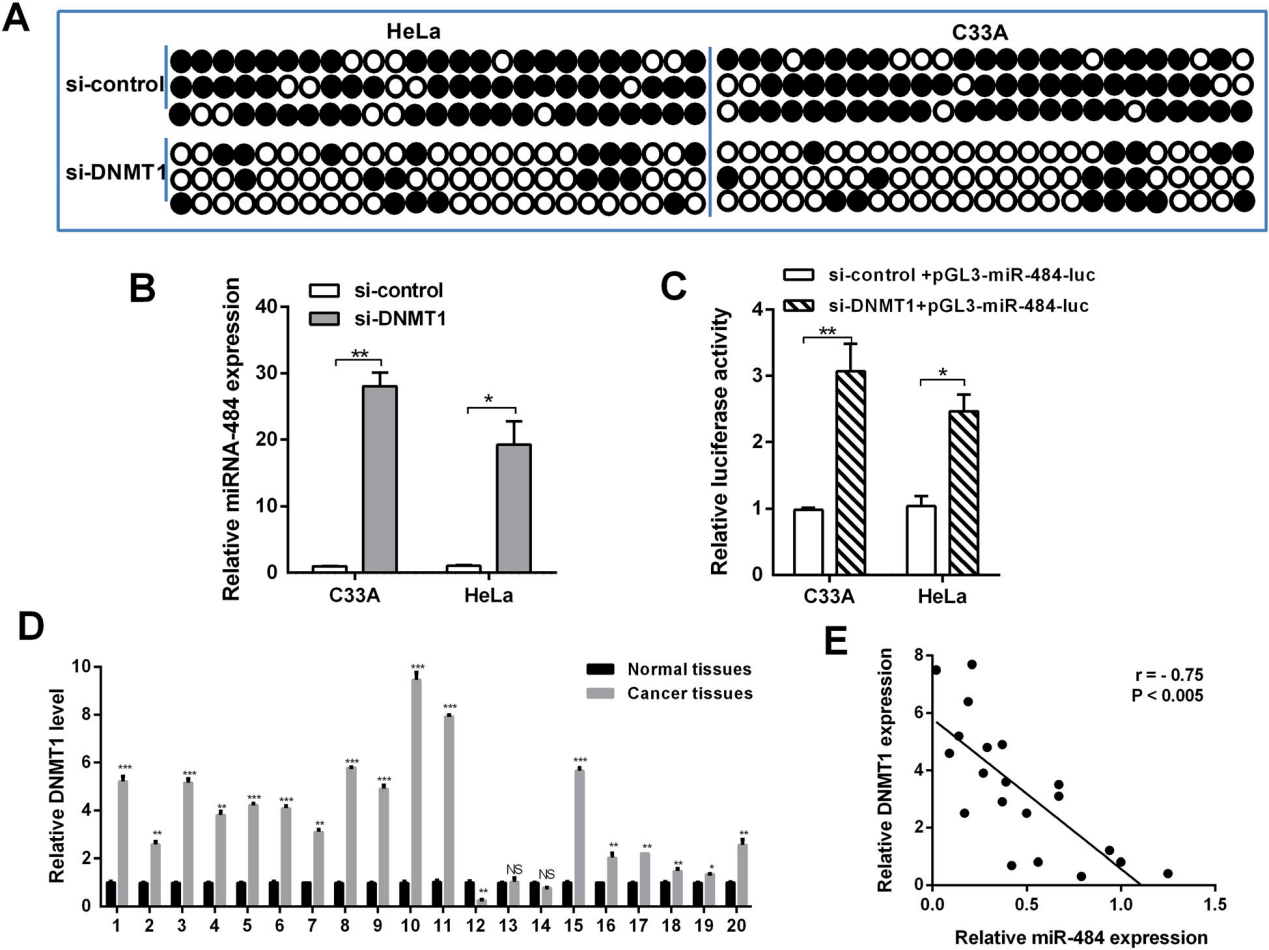
The correlation between DNMT1 downregulation and miR-484 expression. **a** Genomic bisulfite sequencing revealed the methylation status of CC cells transfected with DNMT1 siRNA and control siRNA. **b** and **c** The mRNA level and the promoter activity of miR-484 after knockdown of DNMT1 in CC cells were determined by RT-qPCR (**b**) and luciferase reporter assay (**c**). **d** The mRNA level of DNMT1 in 20 pairs of CC tissues was measured by RT-qPCR; actin was used for normalization. **e** The correlation between miR-484 and DNMT1 expression. Error bars indicate the mean ± SD of three independent experiments. *p < 0.05, **p < 0.01,***p < 0.001

Because the miR-484 promoter is hypermethylated in CC, we hypothesized that the deregulation of a specific methylase or demethylase induces this process. To identify the putative methylase/demethylase responsible for miR-484 methylation, siRNAs of DNMT1, DNMT3A, DNMT3B, KDM2A, KDM4A, and KDM4B were transfected into HeLa cells respectively (Additional file 1: Figure S2). A detailed analysis by bisulfite sequencing indicated that only the knockdown of DNMT1 significantly reduced the number of methylated CpG sites (Fig. 2a). Therefore, we hypothesize that DNMT1 is involved in the DNA methylation-mediated silencing of miR-484. Indeed, the mRNA level and promoter activity of miR-484 was recovered when DNMT1 was downregulated in CC cells (Fig. 2b and c). We also examined the expression level of DNMT1 in 20 pairs of clinical CC tissues. The results revealed that DNMT1 was generally expressed at a higher level (Fig. 2d). Spearman’s rank correlation analysis revealed a negative correlation between DNMT1 and miR-484 expression (Fig. 2e). The above results suggested that DNMT1 could directly induce the hypermethylation of miR-484 downregulating its expression.

**Fig. 3:**
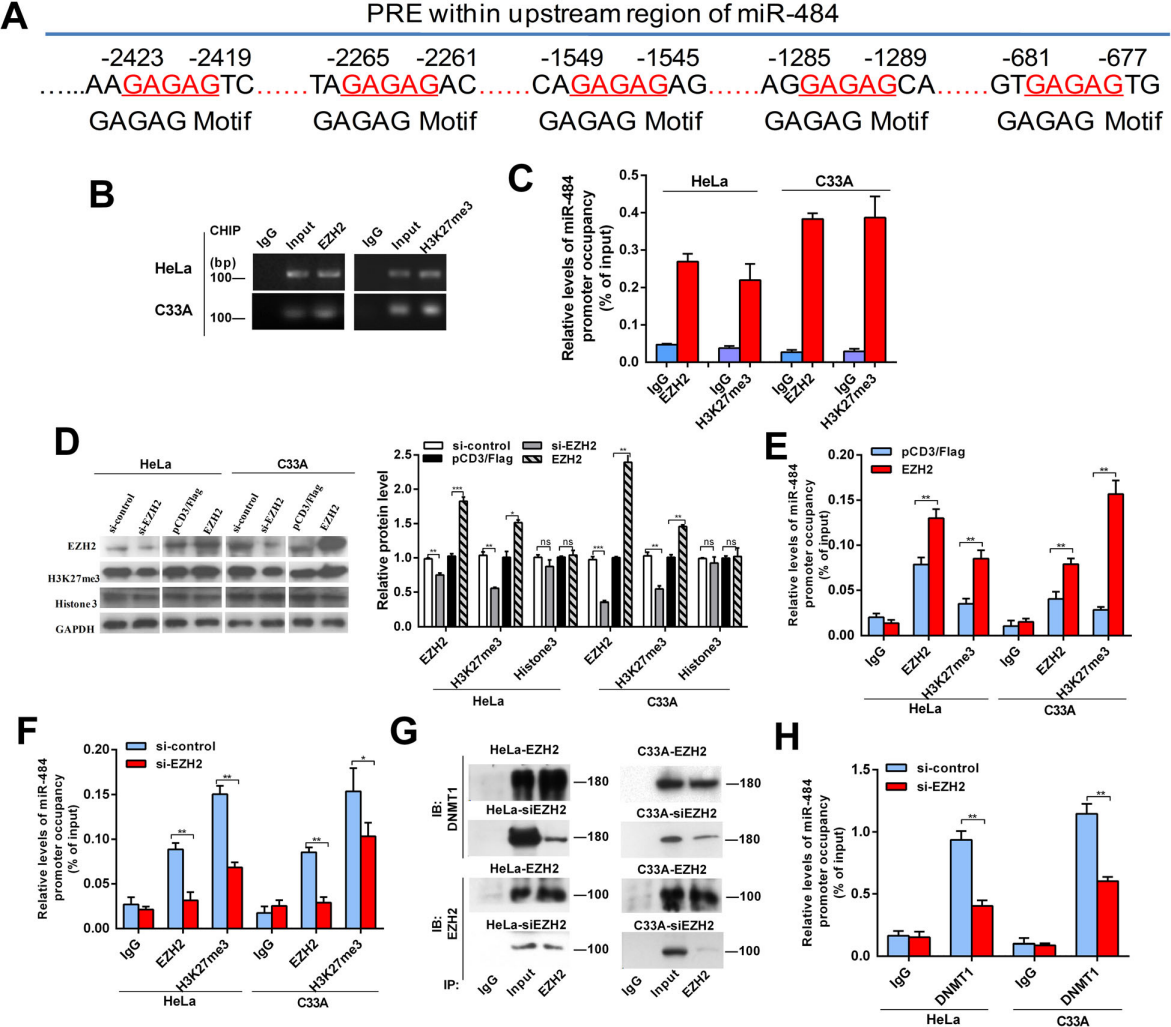
miR-484 is repressed by hypermethylation mediated by EZH2-recruited DNMT1. **a** Schematic location of the core DNA motif (red) of the Polycomb response element (PRE). **b**, **c** PCR (**b**) and CHIP-qPCR (**c**) assay for anti-EZH2 and H3K27me3 in CC cells. **d** Western blot analysis of EZH2, H3K27me3, and Histone3 in CC cells with EZH2 overexpression or downregulation or control. **e**, **f** CHIP-qPCR assays for CC cells after EZH2 modification. **g** Physical interactions between EZH2 and DNMT1 were examined via a Co-IP assay in CC cells with EZH2 overexpression or downregulation. **h** CHIP-qPCR assays for anti-DNMT1 in CC cells after EZH2 downregulation. Mean (*n* = 3) ± SD. Student’s *t* test, *p < 0.05, **p < 0.01

Moreover, we analyzed the upstream and coding region of miR-484 and identified consensus polycomb response elements on the upstream region (Fig. 3a). As the polycomb repressive complex 2 (PRC2) complex has been shown to recruit DNMTs to methylate target genes resulting in transcription repression [20, 21], we deduced that PCR2 may be involved in the repression of miR-484. In addition, enhancer of zeste 2 polycomb repressive complex 2 subunit (EZH2), the core unit of PRC2, can catalyze the histone H3 lysine 27 trimethylation (H3K27me3) [20, 22]. Our results showed that EZH2 bound to the upstream region of miR-484, while high level of the repressive histone methylation marker H3K27me3 was also observed (Fig. 3b and c). Moreover, overexpression of EZH2 increased the levels of H3K27me3 and the occupancies of EZH2 in miR-484 upstream region, while knockdown of EZH2 decreased H3K27 trimethylation and the bindings of EZH2 to the upstream region of the miR-484 locus (Fig. 3d–f).

Furthermore, we evaluated whether EZH2 is responsible for DNMT1 binding to the miR-484 upstream region. Co-IP assays showed that EZH2 physically recruited DNMT1 in CC cells, while the recruitment was reduced obviously after knockdown of EZH2 (Fig. 3g). CHIP assays proved the binding of DNMT1 and demonstrated that EZH2 inhibition led to a remarkable reduction of DNMT1 occupancies in the upstream region of miR-484 (Fig. 3h). These data suggest that EZH2-recruited DNMT1 mediates the hypermethylation and thereby silencing of miR-484.

### miR-484 inhibits CC cell adhesion by regulating the β1-integrin signaling pathway in vitro and tumor growth in vivo

**Fig. 4:**
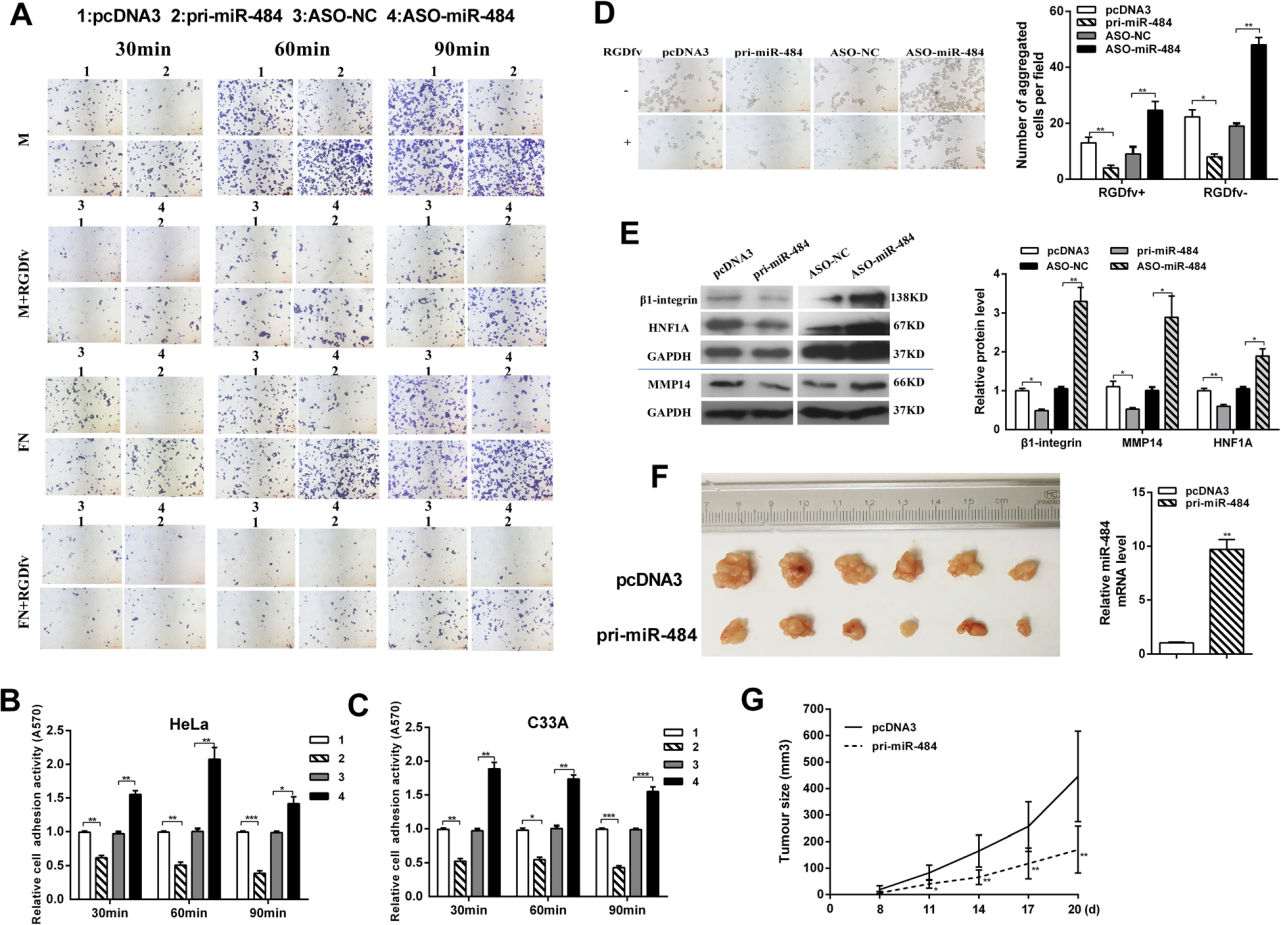
miR-484 suppresses CC cell adhesion in vitro and metastasis in vivo. **a** Cell–matrix adhesion assay of HeLa cells adhering to FN and Matrigel (M) with or without RGDfv for 30, 60, and 90 min, respectively. Representative images under microscope were showed (× 100). **b**, **c** Quantitative results of three independent experiments of cell–matrix adhesion assay in HeLa (**b**) and C33A cells (**c**). The adherent cells were measured by software of ImageJ (*p < 0.05, **p < 0.01). **d** Cell–cell adhesion assay of HeLa cells suspension cultured with or without RGDfv for 24 h. Left: representative images, the photomicrographs were taken at × 100 magnification. Right: quantitative results of three independent experiments (*p < 0.05, **p < 0.01). **e** Western blot results showing β1-integrin, HNF1A, and MMP14 expression in HeLa cells after transfection with pri-miR-484 or ASO-miR-484. The expression levels were normalized to GAPDH (*p < 0.05, **p < 0.01). **f** A nude mouse tumor xenograft model was utilized to study the effect of miR-484 on tumor growth in vivo. The mice were euthanized, and the tumors were isolated 20 days after implantation. The miR-484 level in tumors was shown on the right of the picture. **g** The tumor size was measured 8 days after injection every other day; the tumor growth curve is shown. The tumor volume = length × width^2^ × 1/2, *p < 0.05, **p < 0.01, *n* = 6 (Student’s *t* test)

Adhesion to a target tissue is considered to be one of the most critical steps in the invasive process for metastatic tumor cells. We investigated whether miR-484 can also affect CC cell adhesion. Cell–matrix adhesion assay showed that miR-484 inhibited while ASO-miR-484 promoted cell adhesion to both fibronectin (FN) and Matrigel (M). Adhesion activity of HeLa cells adhering to Matrigel wells was reduced by approximately 38.6%, 49.6%, and 61.8% at 30 min, 60 min, and 90 min, respectively, after miR-484 overexpression (Fig. 4a and b). Similar results were observed in C33A cells (Fig. 4c). The results of cell–cell adhesion assay showed that after overexpression of miR-484, HeLa cells were less aggregated in suspension cultured with or without cell adhesion inhibitor RGDfv (Fig. 4d). When miR-484 was blocked, the number of aggregated HeLa cells was increased compared with control. Next, we detected the protein level of β1-integrin in CC cells, which is known to be associated with cell–matrix adhesion. Interestingly, the protein level of β1-integrin was decreased after transfected with pri-miR-484 (Fig. 4e). These results indicate that miR-484 may affect CC cell adhesion by regulating the β1-integrin signaling pathway.

To determine the effect of miR-484 on CC tumor growth, xenograft tumor experiments were performed in mice. The average volume of tumors derived from HeLa cells was lower in the miR-484 group than in the control group (Fig. 4f and g). In short, miR-484 functions as an anti-oncogene to suppress malignancy in vivo and in vitro.

### miR-484 downregulates MMP14 and HNF1A expression by binding their 3′UTRs in CC cells

**Fig. 5:**
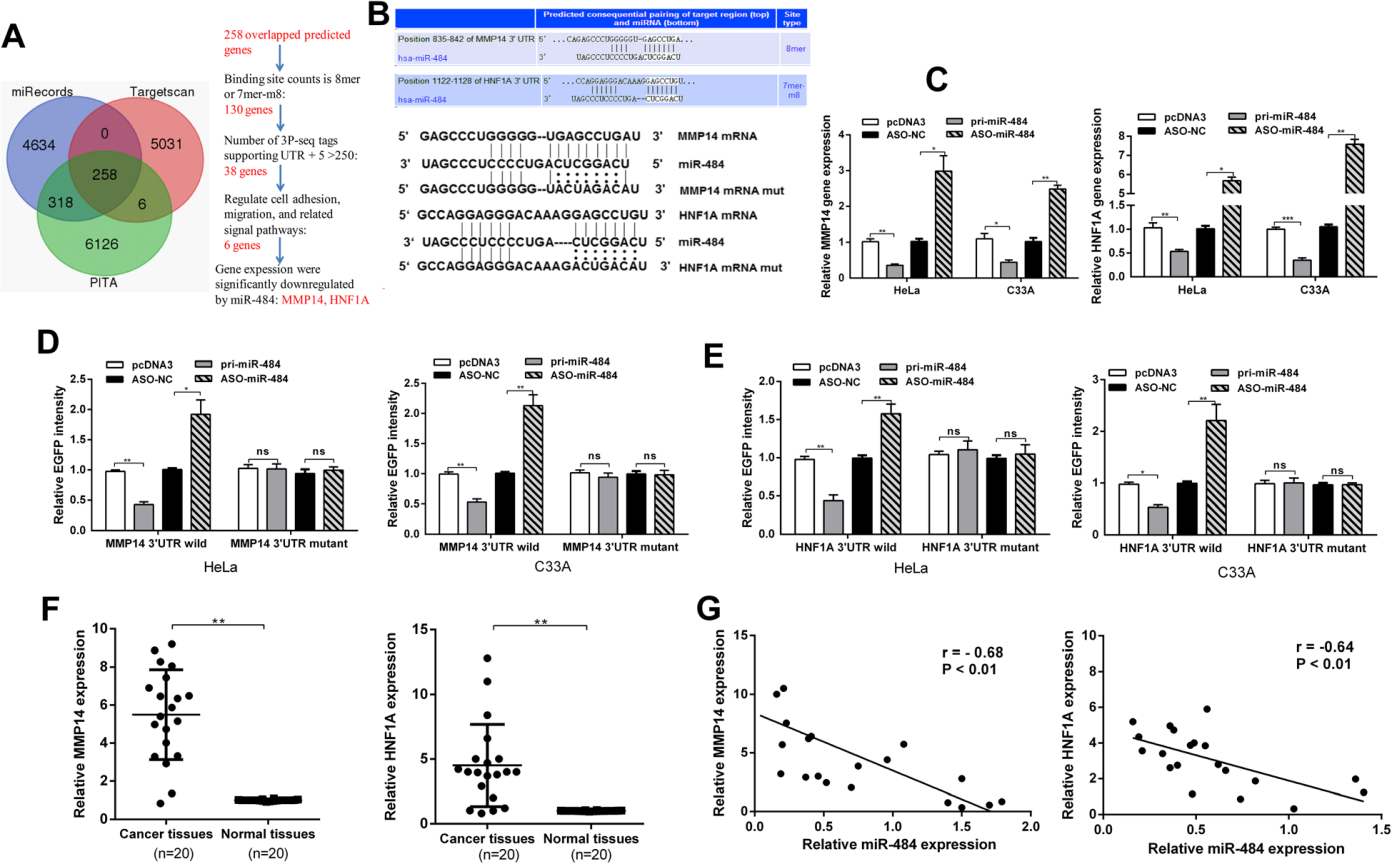
miR-484 suppresses MMP14 and HNF1A expression by targeting their 3′UTRs in CC cells. **a** The diagram shows the potential target genes (overlapping fraction) that were shared by all three databases and the technical route of selecting candidate targets. **b** The predicted miR-484-binding sites in HNF1A and MMP14 mRNA using Targetscan 7.0 are shown. **c** RT-qPCR showing HNF1A and MMP14 mRNA levels after transfection with pri-miR-484 or ASO-miR-484. **d** HeLa and C33A cells were co-transfected with wild-type pcDNA3/EGFP-MMP14 3′UTR or 3′UTR-mut and pri-miR-484 or ASO-miR-484. **e** HeLa and C33A cells were co-transfected with wild-type pcDNA3/EGFP-HNF1A 3′UTR or 3′UTR-mut and pri-miR-484 or ASO-miR-484. **f** RT-qPCR showing the expression of HNF1A and MMP14 in 20 pairs of human cervical cancer tissues and the adjacent non-cancerous tissues. U6 snRNA was used as the internal control. **g** Pearson’s correlation analysis indicated the negative correlation between the expression of miR-484 and HNF1A (*r* = − 0.64**) and MMP14 (*r* = − 0.68**). All of the experiments were repeated three times. *p < 0.05, **p < 0.01, ***p < 0.001; NS, not significant

Our previous work showed that miR-484 suppresses cell migration, invasion, and epithelial– mesenchymal transition (EMT) [10]. In this paper, as miR-484 suppresses cervical cancer cell adhesion, it is important to understand which targets are directly responsible for this phenotype. We used three prediction algorithms (TargetScan, miRecords, and PITA) to predict the targets for miR-484 in common. There were 258 candidate targets shared by all the three databases (the overlapping fraction) (Fig. 5a). Among the potential targets, we selected 6 genes that involved in cell adhesion, migration, and signaling pathways for primary RT-qPCR tests. The results showed that the expression of MMP14 and HNF1A was significantly downregulated by pri-miR-484 (Additional file 1: Figure S3). Therefore, we chose these two genes for further study. The technical route is shown in Fig. 5a.

Next, we further determined the effect of miR-484 on endogenous MMP14 and HNF1A expression. RT-qPCR showed that MMP14 and HNF1A mRNA levels were decreased in HeLa and C33A cells transfected with pri-miR-484 while increased when transfected with ASO-miR-484 (Fig. 5c). Furthermore, Western blotting assays showed a similar relationship: miR-484 decreased while ASO-miR-484 increased MMP14 and HNF1A expression (Fig. 4c). These results indicate that miR-484 suppresses MMP14 and HNF1A expression at both the mRNA and protein levels in CC cells.

To confirm whether MMP14 and HNF1A are direct targets of miR-484 in human CC cells, we performed an EGFP reporter assay using EGFP reporter vectors containing either the wild-type 3′UTR or a mutant 3′UTR with a mutation in the complementary seed sequence of MMP14 and HNF1A (Fig. 5b). In HeLa and C33A cells, miR-484 significantly decreased the intensity of pcDNA3/EGFP-MMP14 3′UTR and pcDNA3/EGFP-HNF1A 3′UTR (Fig. 5d and e). However, EGFP intensity with the mutant 3′UTR of MMP14 or HNF1A was not influenced by either overexpression or inhibition of miR-484 (Fig. 5d and e). These results suggest that miR-484 may directly target MMP14 and HNF1A and downregulates their expression. Therefore, miR-484 regulated cell adhesion and tumor growth through both directly targeting MMP14 and HNF1A.

To further explore the expression levels of MMP14 and HNF1A in cervical cancer, we performed RT-qPCR analyses to examine their expression levels in 20 pairs of CC tissues and adjacent non-tumor tissues as well as a panel of cervical cancer cell lines. We found that MMP14 and HNF1A were upregulated in cancer tissues compared with the adjacent non-tumor tissues (Fig. 5f). Accordingly, we analyzed correlation between miR-484 and MMP14/HNF1A. miR-484 presented a reverse correlation with the expression of MMP14 mRNA (*r* = − 0.68, *p* < 0.01) and HNF1A mRNA (*r* = − 0.64, *p* < 0.01) (Fig. 5g). The results further demonstrated that MMP14 and HNF1A were upregulated in cervical cancer tissues compared with the normal cervix.

### MMP14 and HNF1A promote CC cell adhesion, migration, invasion, and EMT

**Fig. 6:**
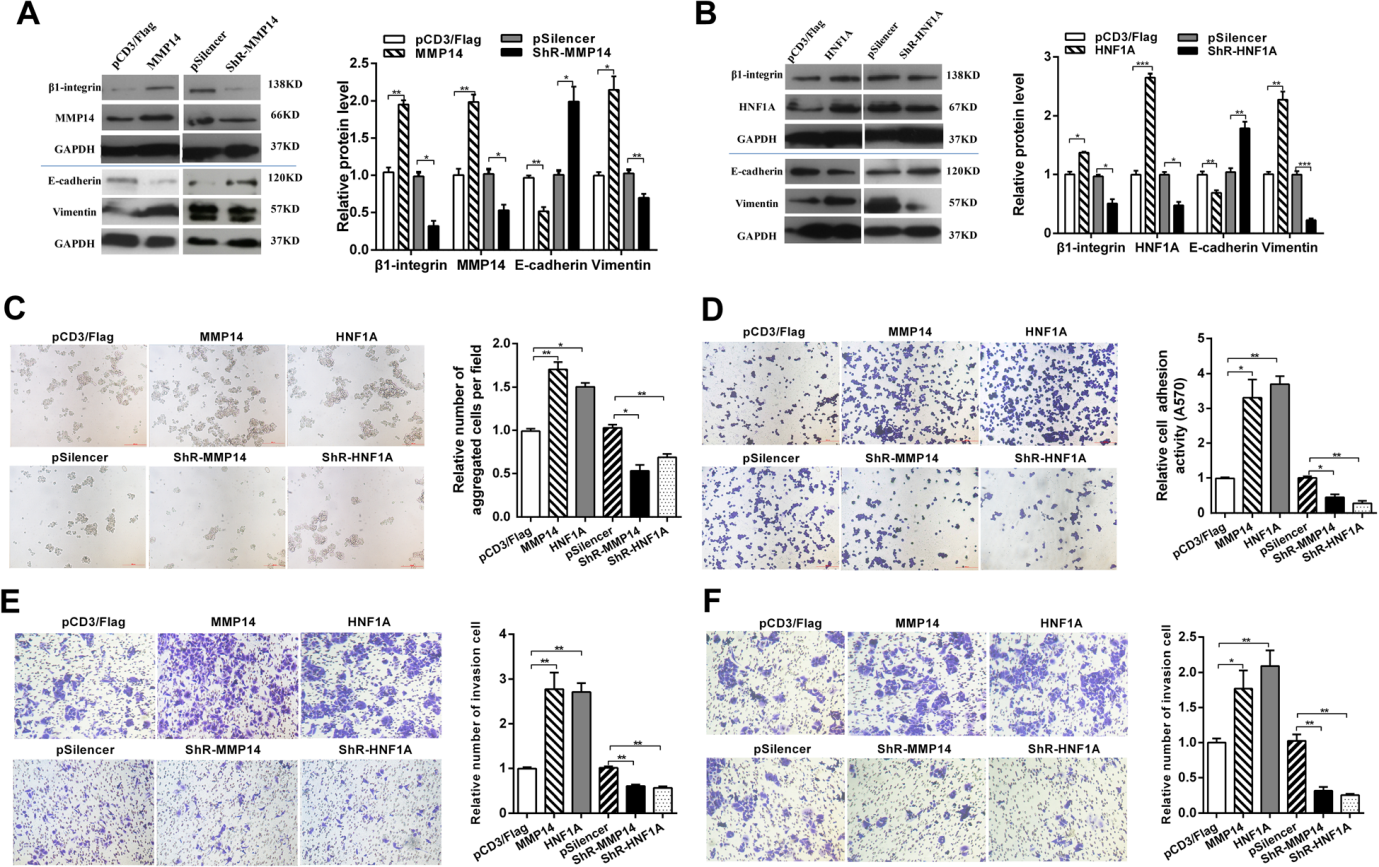
MMP14 and HNF1A promotes CC cell adhesion, migration, and invasion. **a** and **b** The influence of MMP14 (**a**) and HNF1A (**b**) on β-integrin, E-cadherin, vimentin protein expression in HeLa cells was determined by Western blot. **c** and **d** Cell–cell adhesion assay (**c**) and cell–matrix adhesion assay (**d**) of HeLa cells after transfection with pFlag-MMP14/HNF1A or shR-MMP14/HNF1A. **e** and **f** Transwell migration (**e**) and invasion assays (**f**) of HeLa cells after transfection with pFlag-MMP14/HNF1A or shR-MMP14/HNF1A. All of the experiments were repeated three times. *p < 0.05, **p < 0.01, ***p < 0.001

It has been reported that MMP14 functions as an oncogene and enhances the migration and adhesion induced by β-integrin in various cancer cells [23]. Recent studies have showed that HNF1A is a novel oncogene that regulates human pancreatic cancer stem cell properties [24]. To address the metastasis-associated features of CC cells regulated by MMP14 and HNF1A, we constructed both the MMP14 and HNF1A overexpression vector (pFlag-MMP14/HNF1A) and the knockdown plasmid (shR-MMP14/HNF1A), and conducted functional assays (Fig. 6a–f). Western blot assays validated the efficiency of pFlag-MMP14/ HNF1A and shR-MMP14/HNF1A (Fig. 6a and b).

The cell–cell adhesion and cell–matrix adhesion assays in HeLa cells showed that MMP14 or HNF1A overexpression significantly increased cell–cell adhesion and cell–matrix adhesion, whereas MMP14 or HNF1A downregulation repressed the HeLa cell–cell adhesion and cell–matrix adhesion (Fig. 6c and d). Cell–matrix adhesion assays indicated that adhesion activities of MMP14- and HNF1A-overexpressed HeLa cells were, respectively, increased by 3.3- and 3.7-folds compared with the control groups. In the contrary, MMP14- and HNF1A- repressed HeLa cells showed 44.3% and 27.3% adhesion activities compared with the control (Fig. 6d). We also assessed the influence of MMP14 and HNF1A on β1-integrin. As shown in Fig. 6a and b, β1-integrin expression was enhanced by overexpression of MMP14 or HNF1A compared with the control group. These results indicated that MMP14 and HNF1A promote cell adhesion by regulating the β1-integrin signaling pathway.

In addition, transwell migration and invasion assays showed that overexpression of MMP14 and HNF1A significantly increased the migration ability by approximately 2.8- and 2.7-folds and the invasion ability by 1.8- and 2.1-folds in HeLa cells respectively (Fig. 6e and f). Knockdown of MMP14 and HNF1A decreased the migration ability by 40% and 44% and the invasion ability by 68% and 75% in HeLa cells respectively (Fig. 6e and f). Because MMP14 and HNF1A significantly promote the migration and invasion, we tested whether MMP14 and HNF1A affected the expression of key molecular markers of EMT, including E-cadherin and vimentin. Western blot analysis showed that overexpression of MMP14 and HNF1A decreased E-cadherin protein levels but increased vimentin protein levels in HeLa cells (Fig. 6a and b). Taken together, these results indicated that MMP14 and HNF1A promote cell migration and invasion and have effect on EMT process.

### MMP14/HNF1A overexpression partly rescues the suppression of the malignant behavior induced by miR-484 in CC cells

**Fig. 7:**
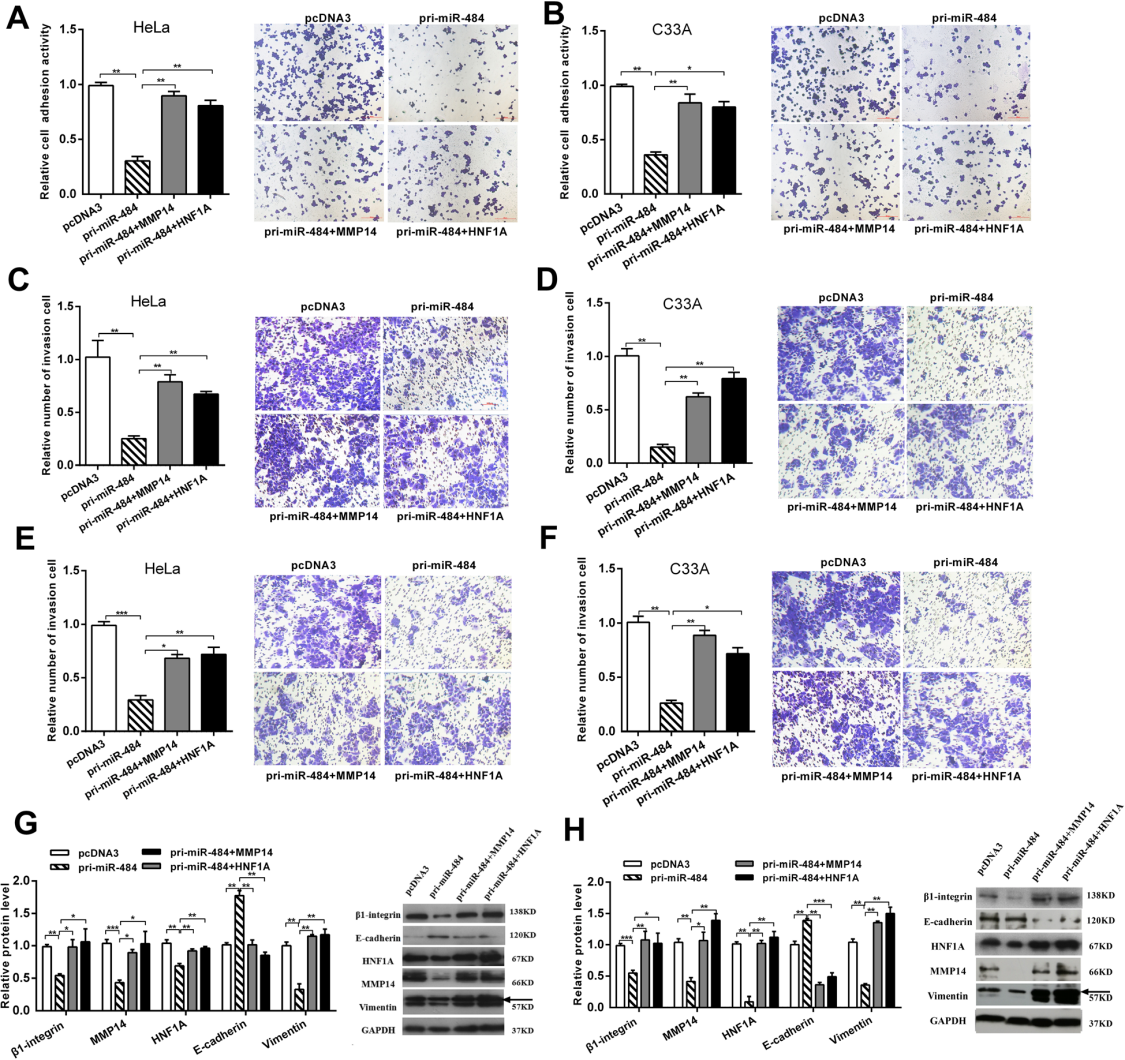
MMP14/HNF1A overexpression partly rescues the suppression of the malignant behavior induced by miR-484 in CC cells. Co-transfection with pri-miR-484 and pFlag-MMP14 (MMP14) or pFlag-HNF1A (HNF1A) showed that overexpression of MMP14 or HNF1A partly rescued cell adhesion (**a** and **b**), migration (**c** and **d**), and invasion (**e** and **f**) in HeLa and C33A cells. **g** and **h** Western blot showed the protein levels of β-integrin, E-cadherin, and vimentin after transfection with pri-miR-484 and MMP14/HNF1A. All of the experiments were repeated three times. *p < 0.05, **p < 0.01. ***p < 0.001

To demonstrate that miR-484 may function as an anti-oncogene by directly regulating MMP14 and HNF1A, we performed rescue versions of the adhesion, migration, invasion, and western blot experiments in HeLa and C33A cells. First, we co-transfected miR-484 with the MMP14 or HNF1A expression plasmid without the 3′UTR which has been proved worked (Fig. 6a and b) and confirmed the overexpression of MMP14 or HNF1A could rescue the decrease in MMP14 or HNF1A protein levels caused by miR-484 (Fig. 7g and h). Then, we performed a series of functional rescue experiments. As expected, the restoration of MMP14 or HNF1A expression mostly blocked the inhibitory effects of miR-484 on the cell adhesion (Fig. 7a and b), cell migration (Fig. 7c and d), and cell invasion (Fig. 7e and f). Meanwhile, the ectopic expression of MMP14 or HNF1A counteracts the inhibition of EMT and β1-integrin expression induced by miR-484 in CC cells (Fig. 7g and h).

### miR-484 negatively regulates the WNT/MAPK and TNF signaling pathway in CC cells

**Fig. 8:**
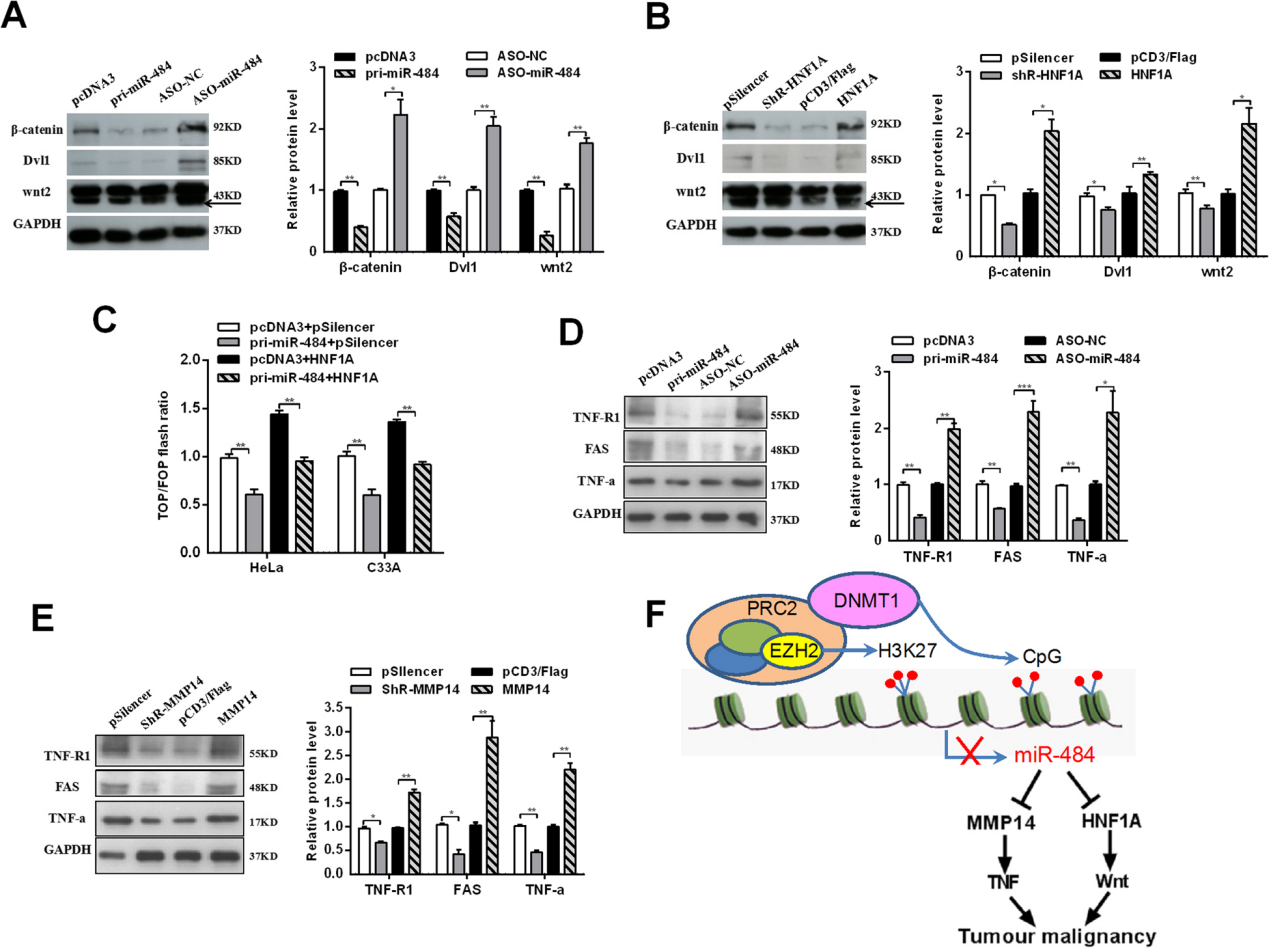
miR-484 negatively regulates the WNT/MAPK and TNF signaling pathway in CC cells. **a** and **d** Western blot were used to measure the protein levels after transfection with pri-miR-484 or ASO-miR-484. **b** Western blot showed the protein levels in cells treated with pFlag-HNF1A (HNF1A) or shR-HNF1A. **c** HeLa and C33A cells were co-transfected with pTOP/flash or pFOP/flash, pcDNA3, pri-miR-484, pFlag-HNF1A, or pri-miR-484 and pFlag-HNF1A, respectively, after which luciferase reporter assays were performed. **e** Western blot showed the protein levels in cells treated with pFlag-MMP14 (MMP14) or shR-MMP14. All of the experiments were repeated three times. *p < 0.05, **p < 0.01, ***p < 0.001. **f** The model by which DNMT1 epigenetically mediates miR-484 repression with resultant MMP14/HNF1A activation, leading to tumor malignancy in CC

It has been reported that HNF4a inhibits HCC progression by regulating the Wnt/β1-catenin signaling pathway [25, 26]. To investigate the influence of miR-484 on WNT signaling pathway through targeting HNF1A, Western blot assays were utilized to detect downstream effectors of WNT signaling in CC cells transfected with miR-484 and HNF1A (Fig. 8a and b). In both HeLa and C33A cells, the expression of wnt2, Dvl1, and β1-catenin, downstream effectors of the WNT/MAPK pathway, were significantly reduced by miR-484 overexpression but increased by HNF1A restoration. And TOP/FOP luciferase reporter assays were also performed (Fig. 8c), which is a common WNT pathway activation reporter assay [27, 28]. The results showed that miR-484 weakened but HNF1A enhanced TOP/FOP flash ratio (Fig. 8c).

Previous studies suggested that MMP14 is a promoter of the TNF signaling pathway, its silencing results in a better treatment of patients resistant to anti-TNF therapy [29]. We also detected the influence of miR-484 and MMP14 on the TNF signaling pathway (Fig. 8d and e). The results of Western blot showed that the expression of TNF receptor 1 (TNF-R1), fatty acid synthase (FAS), and TNF-α, downstream effectors of the TNF pathway, were reduced by miR-484 restoration and significantly increased by miR-484 depletion (Fig. 8d). While, MMP14 showed the contrary result. The expression of TNF-R1, FAS, and TNF-α were increased by MMP14 overexpression while decreased by MMP14 downregulation (Fig. 8e). These results indicate that miR-484 modulates the WNT/MAPK and TNF signaling pathway in a HNF1A- or MMP14-dependent manner, respectively, in CC cells.

## Discussion

Our current study provides novel evidence that epigenetic silencing of miR-484 is the specific event leading to metastasis in CC. Previous work showed that miR-484 was downregulated both in vivo and in vitro of CC, but the mechanism is still not understood. In this study, we found that downregulation of miR-484 was associated with miR-484 locus hypermethylation in metastatic CC. The methylation level of miR-484 decreased after 5-Aza-CdR treatment. Furthermore, miR-484 was epigenetically silenced by EZH2-recruited DNMT1 and suppressed CC cell metastasis and EMT by targeting MMP14 and HNF1A. MMP14 and HNF1A promoted cell adhesion by regulating the β1-integrin signaling pathway as well as cell migration and invasion by regulating the EMT process. Overexpression of either of the two targets could partly rescue the suppression of the malignant behavior induced by miR-484. Additionally, miR-484 modulates the WNT/MAPK and TNF signaling pathway in a HNF1A- or MMP14-dependent manner, respectively, in CC cells. These new findings reveal a critical DNMT1/miR-484/MMP14/HNF1A cascade for the promotion of CC metastasis, suggesting the application of miRNA as a new prognostic marker and therapeutic strategy for patients with, or at risk of, metastasis (Fig. 8f).

DNA methylation is a reversible enzyme-mediated modification involved in embryonic and stem cell development and cancer progression [30]. DNMTs catalyze the transfer of a methyl group from S-adenosylmethionine (AdoMet) to the 5-position of cytosine in DNA, and the specific pattern of methylation provides differential accessibility to the DNA information code by affecting DNA–protein interactions and chromatin condensation, and DNMT1 is best known as the maintenance methyltransferase that copies methylation patterns after DNA replication [31]. EZH2, a catalytic subunit of the PCR2 complex, serves as a recruitment platform for DNMTs [32, 33]. In the current study, we reported that EZH2-recruited DNMT1 occupied the upstream region of miR-484 and inhibited miR-484 expression through DNA methylation. Previous studies showed that the expression of DNMT1 is higher in CC tissues compared with that in normal tissues and its higher expression is significantly associated with poor survival outcomes [33]. Consistently, we found that DNMT1 was overexpressed in CC cells and a negative correlation between DNMT1 and miR-484 expression was also observed. Our study provides new insight into the mechanism of DNMT1 regulating miR-484 expression and subsequent HNF1A and MMP14 expression and its contribution to CC cell metastasis.

It is well known that miRNAs negatively regulate gene expression by interacting with 3′UTR of target gene mRNA [34]. After intersecting analysis with bioinformatics algorithms and RT-qPCR verification, we identified that HNF1A and MMP14 served as direct targets of miR-484. Further analysis confirmed that miR-484 overexpression inhibited HNF1A and MMP14 expression at both the mRNA and protein levels. As reported, MMP14, an important cell adhesion molecule involved in the EMT process, contributes to invasion, metastasis, and angiogenesis through ECM degradation [11]. As a transcriptional factor, HNF1A has been shown to affect intestinal epithelial cell growth and cell lineages differentiation [35, 36]. However, it seems that HNF1A showed different functions in different cancers. Previous studies in other human cancers have suggested a tumor suppressor role of the HNF1A gene. Whereas, in pancreatic cancer, HNF1A is a novel oncogene that regulates human stem cell properties [24]. Our study showed that MMP14 and HNF1A promote CC cell adhesion by regulating the β1-integrin signaling pathway and increased migration and invasion by regulating the EMT process. Moreover, our results verified that MMP14/HNF1A overexpression partly rescues the inhibitory effects of miR-484 on CC cell adhesion, migration, invasion, and EMT, thus supporting the contribution of miR-484-regulated MMP14/HNF1A to CC metastasis and EMT. It is noticeable that this is the first report discussing a potential role for HNF1A in CC cells.

In addition, our results demonstrated that miR-484 modulates the WNT/MAPK signaling pathway in a HNF1A-dependent manner. The effects of miR-484 in CC metastasis may be mediated by inhibiting the WNT/MAPK pathway to induce G1/S arrest and inhibit the EMT process. WNT pathways, which direct growth and patterning during embryonic development, are indispensable for development and have been implicated in tumorigenesis as a potential therapeutic target against cancer [37, 38]. Tumor necrosis factor (TNF) is characterized by its anti-tumor performance in cancer cells. As a member of TNF superfamily of cytokines, TNF mediates cell processes such as inflammation, differentiation, proliferation, and apoptosis [39]. More specifically, it has been proved that MMP14 is a promoter of the TNF signaling pathway [40]. Our results indicated that miR-484/MMP14 suppressed proliferation and invasion of CC cells by inhibiting TNF signaling pathway.

Due to the important roles in multiple cancer processes, miRNAs have emerged as therapeutic targets in many cancers [41]. Because of the epigenetically silencing of some suppressive miRNAs, reversion of their expression should be a valid therapeutic method for cancer therapy. Lots of studies have showed that ectopic expression of miRNAs display anti-cancer effects [6, 22, 41]. Similarly, we found that restoration of miR-484 repressed tumor growth in a mouse model. These findings indicate a new therapeutic strategy for CC metastasis through modifying miRNA expression.

## Conclusions

We identify miR-484 as a metastasis specific suppressor with hypermethylation of promoter in CC and reveal a novel pathway in which DNMT1 epigenetically mediates miR-484 repression with resultant MMP14/HNF1A activation, which results in tumor metastasis in CC. Our study also highlights the prognostic value of miR-484 methylation and the potential therapeutic effect of miR-484 in CC metastasis, thereby facilitating the development of novel therapeutic method against CC metastasis.

## Supplementary information

**Additional file 1: Figure S1.** Construction of the pGL3-miR-484-luc vector. **Figure S2.** Screening for the potential enzyme responsible for the hypermethylation of the miR-484 promoter. **Figure S3.** Primary RT-qPCR test of the targets of miR-484. **Table S1.** The primers and oligonucleotides used in this work.

## Data Availability

The datasets analyzed for the current study are available from the corresponding author on reasonable request.

## Abbreviations

5-Aza-CdR: 5-Aza-2-deoxy-cytidine
ASO-miR-484: The 2′-*O*-methyl-modified antisense oligonucleotide of miR-484
ASO-NC: The scramble control oligonucleotides
CC: Cervical cancer
DNMT: DNA methyltransferases enzymes
EMT: Epithelial–mesenchymal transition
HNF1A: The hepatocyte nuclear factor 1α
MBDs: Methyl-CpG-binding domain proteins
miRNAs: microRNAs
MMP: Membrane-bound matrix metalloproteinase

## References

[1] Tjalma WAA (2017). Diagnostic performance of dual-staining cytology for cervical cancer screening: a systematic literature review. Eur J Obstet Gynecol Reprod Biol.

[2] Hu Y, Tang H (2014). MicroRNAs regulate the epithelial to mesenchymal transition (EMT) in cancer progression. Microrna.

[3] Sun Y, Yang X, Liu M, Tang H (2016). B4GALT3 up-regulation by miR-27a contributes to the oncogenic activity in human cervical cancer cells. Cancer Lett.

[4] Guo J, Lv J, Liu M, Tang H (2015). miR-346 up-regulates Argonaute 2 (AGO2) protein expression to augment the activity of other microRNAs (miRNAs) and contributes to cervical cancer cell malignancy. J Biol Chem.

[5] Chen Z, Wang X, Liu R, Chen L, Yi J, Qi B, Shuang Z (2017). KDM4B-mediated epigenetic silencing of miRNA-615-5p augments RAB24 to facilitate malignancy of hepatoma cells. Oncotarget.

[6] Li Z, Wong KY, Calin GA, Chng WJ, Chan GC, Chim CS (2019). Epigenetic silencing of miR-340-5p in multiple myeloma: mechanisms and prognostic impact. Clin Epigenetics.

[7] Jones PA, Baylin SB (2007). The epigenomics of cancer. Cell.

[8] Lujambio A, Ropero S, Ballestar E, Fraga MF, Cerrato C, Setien F, Casado S (2007). Genetic unmasking of an epigenetically silenced microRNA in human cancer cells. Cancer Res.

[9] Esteller M (2007). Cancer epigenomics: DNA methylomes and histone-modification maps. Nat Rev Genet.

[10] Hu Y, Xie H, Liu Y, Liu W, Liu M, Tang H (2017). miR-484 suppresses proliferation and epithelial-mesenchymal transition by targeting ZEB1 and SMAD2 in cervical cancer cells. Cancer Cell Int.

[11] Liu Y, Zhang Y, Wu H, Li Y, Zhang Y, Liu M, Li X (2017). miR-10a suppresses colorectal cancer metastasis by modulating the epithelial-to-mesenchymal transition and anoikis. Cell Death Dis.

[12] Cereghini S, Ott MO, Power S, Maury M (1992). Expression patterns of vHNF1 and HNF1 homeoproteins in early postimplantation embryos suggest distinct and sequential developmental roles. Development.

[13] Haumaitre C, Reber M, Cereghini S (2003). Functions of HNF1 family members in differentiation of the visceral endoderm cell lineage. J Biol Chem.

[14] Hajarnis SS, Patel V, Aboudehen K, Attanasio M, Cobo-Stark P, Pontoglio M, Igarashi P (2015). Transcription factor hepatocyte nuclear factor-1beta (HNF-1beta) regulates microRNA-200 expression through a long noncoding RNA. J Biol Chem.

[15] Kato N, Tamura G, Motoyama T (2008). Hypomethylation of hepatocyte nuclear factor-1beta (HNF-1beta) CpG island in clear cell carcinoma of the ovary. Virchows Arch.

[16] Kim L, Liao J, Zhang M, Talamonti M, Bentrem D, Rao S, Yang GY (2008). Clear cell carcinoma of the pancreas: histopathologic features and a unique biomarker: hepatocyte nuclear factor-1beta. Mod Pathol.

[17] Buchner A, Castro M, Hennig A, Popp T, Assmann G, Stief CG, Zimmermann W (2010). Downregulation of HNF-1B in renal cell carcinoma is associated with tumor progression and poor prognosis. Urology.

[18] Nemejcova K, Cibula D, Dundr P (2015). Expression of HNF-1beta in cervical carcinomas: an immunohistochemical study of 155 cases. Diagn Pathol.

[19] Wan HY, Li QQ, Zhang Y, Tian W, Li YN, Liu M, Li X (2014). MiR-124 represses vasculogenic mimicry and cell motility by targeting amotL1 in cervical cancer cells. Cancer Lett.

[20] Piunti A, Shilatifard A (2016). Epigenetic balance of gene expression by polycomb and COMPASS families. Science.

[21] Hanahan D, Weinberg RA (2011). Hallmarks of cancer: the next generation. Cell.

[22] Vella S, Pomella S, Leoncini PP, Colletti M, Conti B, Marquez VE, Strillacci A (2015). MicroRNA-101 is repressed by EZH2 and its restoration inhibits tumorigenic features in embryonal rhabdomyosarcoma. Clin Epigenetics.

[23] Mori H, Lo AT, Inman JL, Alcaraz J, Ghajar CM, Mott JD, Nelson CM (2013). Transmembrane/cytoplasmic, rather than catalytic, domains of Mmp14 signal to MAPK activation and mammary branching morphogenesis via binding to integrin beta1. Development.

[24] Abel EV, Goto M, Magnuson B, Abraham S, Ramanathan N, Hotaling E, Alaniz AA, et al. HNF1A is a novel oncogene that regulates human pancreatic cancer stem cell properties. Elife. 2018;7. 10.7554/eLife.33947 PMC6122955 30074477

[25] Kwon M, Lee SJ, Wang Y, Rybak Y, Luna A, Reddy S, Adem A (2014). Filamin A interacting protein 1-like inhibits WNT signaling and MMP expression to suppress cancer cell invasion and metastasis. Int J Cancer.

[26] Wu N, Zhang YL, Wang HT, Li DW, Dai HJ, Zhang QQ, Zhang J (2016). Overexpression of hepatocyte nuclear factor 4alpha in human mesenchymal stem cells suppresses hepatocellular carcinoma development through Wnt/beta-catenin signaling pathway downregulation. Cancer Biol Ther.

[27] Gerard B, Tait L, Nangia-Makker P, Shekhar MP (2011). Rad6B acts downstream of Wnt signaling to stabilize beta-catenin: implications for a novel Wnt/beta-catenin target. J Mol Signal.

[28] Jin G, Mizutani A, Fukuda T, Otani T, Yan T, Prieto Vila M, Murakami H (2013). Eosinophil cationic protein enhances stabilization of beta-catenin during cardiomyocyte differentiation in P19CL6 embryonal carcinoma cells. Mol Biol Rep.

[29] Li M, Ren CX, Zhang JM, Xin XY, Hua T, Wang HB, Wang HB (2018). The effects of miR-195-5p/MMP14 on proliferation and invasion of cervical carcinoma cells through TNF signaling pathway based on bioinformatics analysis of microarray profiling. Cell Physiol Biochem.

[30] Bird A (2002). DNA methylation patterns and epigenetic memory. Genes Dev.

[31] Geiman TM, Robertson KD (2002). Chromatin remodeling, histone modifications, and DNA methylation-how does it all fit together?. J Cell Biochem.

[32] Sellers WR, Loda M (2002). The EZH2 polycomb transcriptional repressor—a marker or mover of metastatic prostate cancer?. Cancer Cell.

[33] Piyathilake CJ, Badiga S, Borak SG, Weragoda J, Bae S, Matthews R, Bell WC (2017). A higher degree of expression of DNA methyl transferase 1 in cervical cancer is associated with poor survival outcome. Int J Womens Health.

[34] He L, Hannon GJ (2004). MicroRNAs: small RNAs with a big role in gene regulation. Nat Rev Genet.

[35] Lussier CR, Brial F, Roy SA, Langlois MJ, Verdu EF, Rivard N, Perreault N (2010). Loss of hepatocyte-nuclear-factor-1alpha impacts on adult mouse intestinal epithelial cell growth and cell lineages differentiation. PLoS One.

[36] D’Angelo A, Bluteau O, Garcia-Gonzalez MA, Gresh L, Doyen A, Garbay S, Robine S (2010). Hepatocyte nuclear factor 1alpha and beta control terminal differentiation and cell fate commitment in the gut epithelium. Development.

[37] Taipale J, Beachy PA (2001). The Hedgehog and Wnt signalling pathways in cancer. Nature.

[38] Ghosh N, Hossain U, Mandal A, Sil PC (2019). The Wnt signaling pathway: a potential therapeutic target against cancer. Ann N Y Acad Sci.

[39] Holdbrooks AT, Britain CM, Bellis SL (2018). ST6Gal-I sialyltransferase promotes tumor necrosis factor (TNF)-mediated cancer cell survival via sialylation of the TNF receptor 1 (TNFR1) death receptor. J Biol Chem.

[40] Xu L, Xu Q, Li X, Zhang X (2017). MicroRNA-21 regulates the proliferation and apoptosis of cervical cancer cells via tumor necrosis factor-alpha. Mol Med Rep.

[41] Ortiz I, Barros-Filho MC, Dos Reis MB, Beltrami CM, Marchi FA, Kuasne H, do Canto LM, et al. Loss of DNA methylation is related to increased expression of miR-21 and miR-146b in papillary thyroid carcinoma. Clin Epigenetics 2018;10:144. 10.1186/s13148-018-0579-8 PMC6245861 30454026

