# Supplementary material for: DNMT1 recruited by EZH2-mediated silencing of miR-484 contributes to the malignancy of cervical cancer cells through MMP14 and HNF1A
Source: Clin Epigenetics. 2019 Dec 7;11:186. doi: 10.1186/s13148-019-0786-y (PMC6898970; doi:10.1186/s13148-019-0786-y)
Supplement: Supplementary file 1 — Additional file 1: Figure S1. Construction of the pGL3-miR-484-luc vector. Figure S2. Screening for the potential enzyme responsible for the hypermethylation of the miR-484 promoter. Figure S3. Primary RT-qPCR test of the targets of miR-484. Table S1. The primers and oligonucleotides used in this work. [file 13148_2019_786_MOESM1_ESM.doc]

DNMT1 recruited by EZH2 mediated silencing of miR-484 contributes to the malignancy of cervical cancer cells through MMP14 and HNF1A

Yang Hu^1^, Fuxia Wu^1^, Yankun Liu^2^, Qian Zhao^3^, Hua Tang^1^*

^1^Tianjin Life Science Research Center, Tianjin Laboratory of Inflammation Biology, Collaborative Innovation Center of Tianjin for Medical Epigenetics, Department of Pathogen Biology, Basic Medical School, Tianjin Medical University, Tianjin 300070, China

^2^ The Cancer Institute, Tangshan People's Hospital, Tangshan 063001, China.

^3^ Department of Cell Biology , Tianjin Medical University , Tianjin 300070, China.

^*^Corresponding author: Dr. Hua Tang

Address: 22 Qi-Xiang-Tai Road, Tianjin 300070, China

E-mail address: [htang2002@yahoo.com](mailto:htang2002@yahoo.com); [tangh@tmu.edu.cn](mailto:tangh@tmu.edu.cn). Tel & Fax: +86 22 23542503

**Figure S1.** Construction of the pGL3-miR-484-luc vector. The luciferase reporter assay showed that the fragment that we inserted upstream of the reporter gene had strong promoter activity.


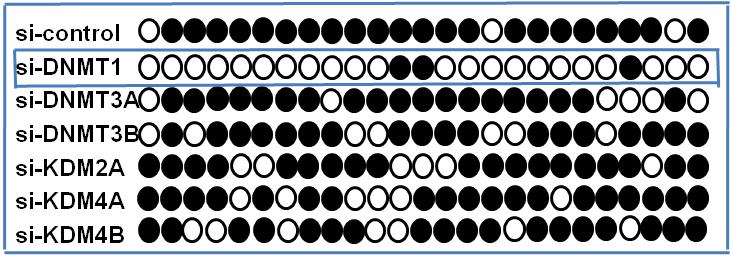


**Figure S2.** Screening for the potential enzyme responsible for the hypermethylation of the miR-484 promoter. siRNAs for the knockdown of DNMT1, DNMT3a, DNMT3b, KDM4A, KDM4B, and KDM6B were transfected into HeLa cell. However, only knockdown of DNMT1 significantly reduced the methylation of CpG loci according to genomic bisulfate sequencing.


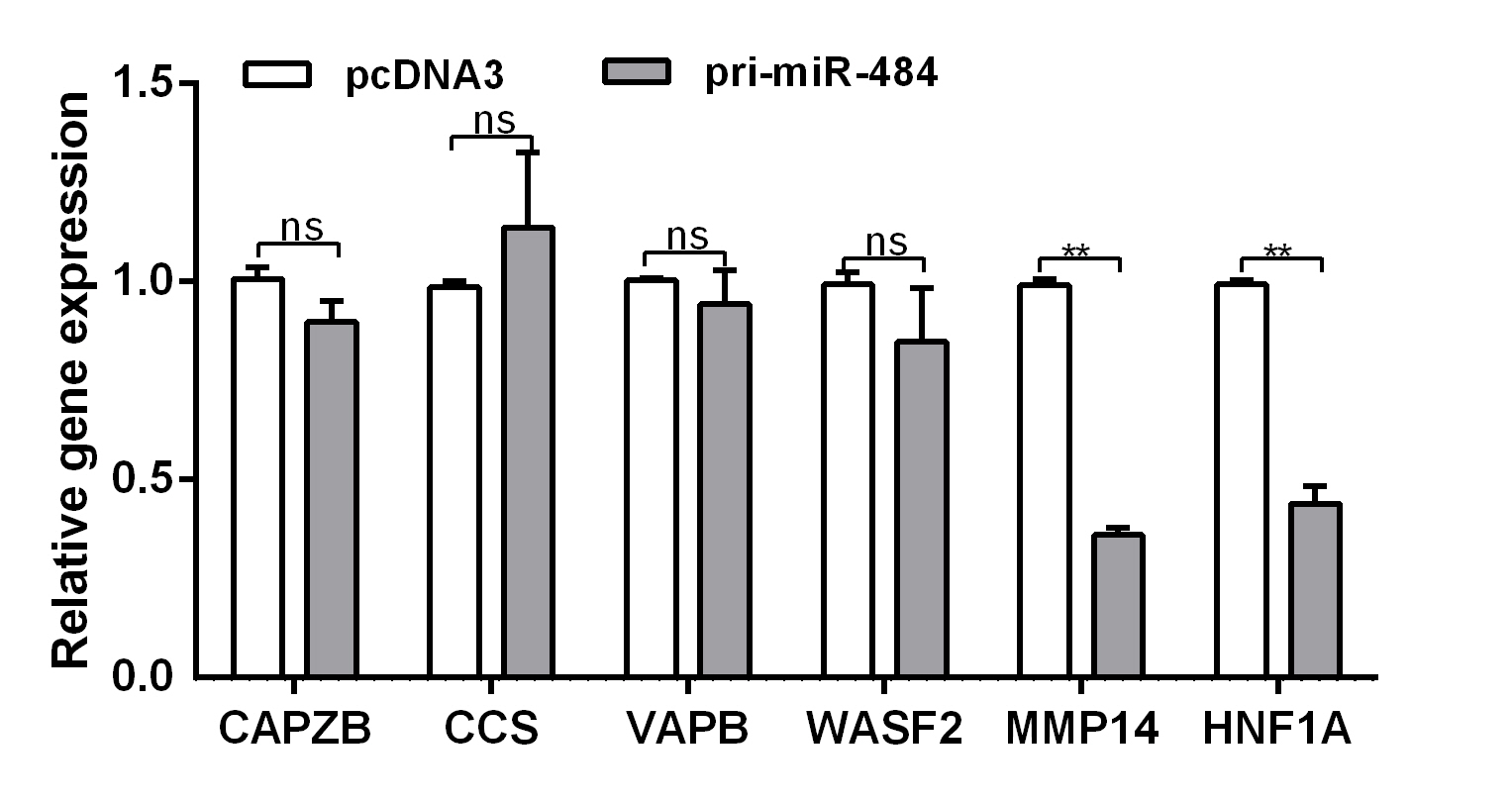


**Figure S3.** Primary RT-qPCR test of the targets of miR-484. RT-qPCR showing 6 predicted genes (CAPZB, CCS, VAPB, WASF2, MMP14 and HNF1A) mRNA levels after transfection with pri-miR-484 in HeLa. The results showed that MMP14 and HNF1A expression were significantly downregulated by pri-miR-484.

**Table 1. The primers and** Oligonucleotides used in this work

| **Name** | **Primer Sequence(5’-3’)** |
| --- | --- |
| **vector constructions** |  |
| EZH2-EcoRI-S | CGGAATTCATGGGCCAGACTGGGAAGA |
| EZH2–XhoI-AS | CCGCTCGAGTCAAGGGATTTCCATTTCTCT |
| MMP14-EcoRI-S | GCGAATTCCGGACCATGTCTCCCGCCCCA |
| MMP14-XhoI -AS | GCACCTCGAGGCGACCTTGTCCAGCAGGGAACG |
| MMP14-shR-S | GATCCGGAAACAAGTACTACCGTTTCCTCGAGGAAACGGTAGTACTTGTTTCCTTTTTGA |
| MMP14-shR-AS | AGCTTCAAAAAGGAAACAAGTACTACCGTTTCCTCGAGGAAACGGTAGTACTTGTTTCCG |
| MMP14-3'UTR-S | GATCCGAGCCCTGGGGGTGAGCCTGATAAGCTTG |
| MMP14-3'UTR-AS | AATTCAAGCTTATCAGGCTCACCCCCAGGGCTCG |
| MMP14-3'UTRmut-S | GATCCGAGCCCTGGGGGTACTAGACATAAGCTTG |
| MMP14-3'UTRmut-AS | AATTCAAGCTTATGTCTAGTACCCCCAGGGCTCG |
| miR-484-promoter-S | GGACAGGTACCCAAATACCATCCACGAAGCAAC |
| miR-484-promoter-AS | TGAGTAGATCTAGGCTGCAGGGCCGCGA |
| HNF1A-EcoRI-S | CCGGAATTCGAGGACGAGACGGACGACGATG |
| HNF1A –XhoI-AS | ACCGCTCGAGACAGAGGAGCTGCCCCACCA |
| HNF1A-shR-S(BamHI) | GATCCGTGTGGCGAAGATGGTCAAGTCTCGAGACTTGACCATCTTCGCCACACTTTTTGA |
| HNF1A-shR-AS(HindIII) | AGCTTCAAAAAGTGTGGCGAAGATGGTCAAGTCTCGAGACTTGACCATCTTCGCCACACG |
| HNF1A-3'UTR-S | GATCCGCCAGGAGGGACAAAGGAGCCTGTAAGCTTG |
| HNF1A-3'UTR-AS | AATTCAAGCTTACAGGCTCCTTTGTCCCTCCTGGCG |
| HNF1A-3'UTRmut-S | GATCCGCCAGGAGGGACAAAGACTGACATAAGCTTG |
| HNF1A-3'UTRmut-AS | AATTCAAGCTTATGTCAGTCTTTGTCCCTCCTGGCG |
| **qRT-PCR** |  |
| miR-484-RT | GTCGTATCCAGTGCAGGGTCCGAGGTGCACTGGATACGACATCGGGAG |
| miR-484-Forward | TGCAGTCAGGCTCAGTCCCC |
| U6-RT | GTCGTATCCAGTGCAGGGTCCGAGGTATTCGCACTGGATACGACAAAATATGGAAC |
| U6-Forward | TGCGGGTGCTCGCTTCGGCAGC |
| Reverse | CCAGTGCAGGGTCCGAGGT |
| qPCR-actin–S | CGTGACATTAAGGAGAAGCTG |
| qPCR-actin–AS | CTAGAAGCATTTGCGGTGGAC |
| qPCR-MMP14–S | GACACCCACTTTGACTCTGCC |
| qPCR-MMP14–AS | GGGAGGTAGTCCTGGGTTGAG |
| qPCR-HNF1A–S | AGCGTCATCGAGACCTTCATCTC |
| qPCR-HNF1A–AS | AACAGGCTTTGCTCCTAGCTCTC |
| DNMT1-qPCR-S | TGGTGATGGTGTGTACCTGC |
| DNMT1-qPCR-A | TTCCGGTAGTGCTCTGGGTA |
| **Primer for ChIP:** |  |
| P1-S | CCGACGCCCTTCTCTCCTCTTC |
| P1-AS | TTTAGGGGTTTATCGGGAGGGG |
| P2-S | GCAATAATCCTGTGAAATAG |
| P2-AS | AAGGCACCTTGATGTAGTGG |
| P3-S | ACCTTCCAGCTGTGCACTTCAC |
| P3-AS | AGAGTTGGCTTGTCTCCCTTTC |
| P4-S | TTGATTCCCGACTCCGCAGC |
| P4-AS | CGAAGAGGAGAGAAGGGCG |
| P5-S | TCAGGCTCAGTCCCCTCCCG |
| P5-AS | TTCCTCGGGTCCACGTCACG |
| **Oligonucleotides** |  |
| siEZH2-1: | GGAUCACCGAGAUGAUAAATT |
| siEZH2-2 | CGGCUUCCCAAUAACAGUATT |
| siDNMT1-1 | GCAGGCGGCUCAAAGAUUUTT |
| siDNMT1-2 | GGAUGAGUCCAUCAAGAAATT |
| siDNMT3A | GCCGUCGACAAGAACACAUTT |
| siDNMT3B | UUGUUGUUGGCAACAUCUGAA |
| siKDM2A | UUUUUCCUUCAAUUUCAUCAU |
| siKDM4A | GAGUUAUCAACUCAAGAUA |
| siKDM4B | GGCAUAAGAUGACCCUCAUTT |
